# Effects of home-based exercise on pre-dialysis chronic kidney disease patients: a randomized pilot and feasibility trial

**DOI:** 10.1186/s12882-017-0613-7

**Published:** 2017-06-17

**Authors:** Koji Hiraki, Yugo Shibagaki, Kazuhiro P. Izawa, Chiharu Hotta, Akiko Wakamiya, Tsutomu Sakurada, Takashi Yasuda, Kenjiro Kimura

**Affiliations:** 1 Department of Rehabilitation Medicine, St. Marianna University School of Medicine Hospital, Kawasaki, Japan; 2 Division of Nephrology and Hypertension, Department of Internal Medicine, St. Marianna University School of Medicine, 2-16-1 Sugao Miyamae-ku, Kawasaki, Kanagawa 216-8511 Japan; 3 Graduate School of Health Sciences, Kobe University, Kobe, Japan; 4 Kichijoji Asahi Hospital, Tokyo, Japan; 5 Japan Community Health care Organization Tokyo Takanawa Hospital, Tokyo, Japan

**Keywords:** Pre-dialysis chronic kidney disease, Aerobic exercise, Resistance training, Physical activity

## Abstract

**Background:**

Only a few research is available on the effects of home-based exercise training on pre-dialysis chronic kidney disease (CKD) patients. Therefore, we aimed to elucidate the effect of home-based exercise therapy on kidney function and arm and leg muscle strength in pre-dialysis CKD patients.

**Methods:**

Thirty-six male stage 3–4 pre-dialysis CKD patients (age, 68.7 ± 6.8 years; estimated glomerular filtration rate (eGFR), 39.0 ± 11.6 ml/min/1.73 m^2^) who were being treated as outpatients were included. The subjects were randomly assigned to an exercise intervention group (Ex group: 18) and a control group (C group: 18). The Ex group wore accelerometer pedometers and were instructed to perform home-based aerobic and resistance exercises, such as brisk walking for 30 min per day, for 12 months. The C group subjects wore accelerometer pedometers but received no exercise therapy guidance; the number of steps covered during normal daily activities was recorded for the C group. The outcome measures were changes in kidney function and handgrip and knee extension muscle strength. Values at the baseline (T1) and 12 months later (T2) were compared.

**Results:**

There were no significant differences in baseline characteristics between the two groups; however, the C group was more physically active than the Ex group. Eight subjects dropped out, and 28 subjects (14 in each group) were included in the final analysis. Physical activity increased significantly only in the Ex group. Grip strength (F = 7.0, *p* = 0.01) and knee extension muscle strength (F = 14.3, *p* < 0.01) were found to improve only in the Ex group. Further, the changes in eGFR were not significantly different between the two groups (F = 0.01, *p* = 0.93).

**Conclusions:**

Home-based exercise therapy for pre-dialysis CKD patients was feasible and improved arm and leg muscle strength without affecting kidney function.

**Trial registration:**

UMIN Clinical Trials Registry (UMIN000005091). Registered 2/15/2011.

## Background

A decline in physical function is observed not only in chronic kidney disease (CKD) patients requiring dialysis but also in pre-dialysis CKD patients [1–4]. We previously reported that the physical function of pre-dialysis CKD patients declines as the disease progresses [4]. Further, CKD patients become increasingly frail as kidney function deteriorates [5]. Observational studies have indicated that physical function decline is a risk factor for poor renal prognosis [6] and poor survival [7] in pre-dialysis CKD patients. Therefore, we believe it is important that pre-dialysis CKD patients receive exercise therapy to improve physical function.

Several of the previous interventional studies on exercise therapy for CKD patients including those not requiring dialysis were center-based, and there were several problems with the implementation of the programs. A study on the feasibility of exercise therapy for pre-dialysis CKD patients conducted by Watson et al. [8] reported a 10% recruitment rate in center-based exercise programs, which is a considerably low figure. Further, supervised exercise therapy is costly. For these reasons, it is important to develop home-based exercise programs [8, 9]. Previous interventional studies have mainly been short-term studies with durations of 8–12 weeks, and very few investigated the safety and effectiveness of long-term exercise over a period of 1 year [9–11]. Many interventional studies focused on aerobic exercise, with only a few including resistance training [9]. Further, no studies used accelerometer pedometers to measure the amount of walking exercise performed.

Therefore, we aimed to elucidate the feasibility, effectiveness and safety of home-based exercise interventions for stage 3–4 pre-dialysis CKD patients over a period of 1 year and used pedometers to quantify the amount of walking exercise performed.

## Methods

### Participants

**Fig. 1:**
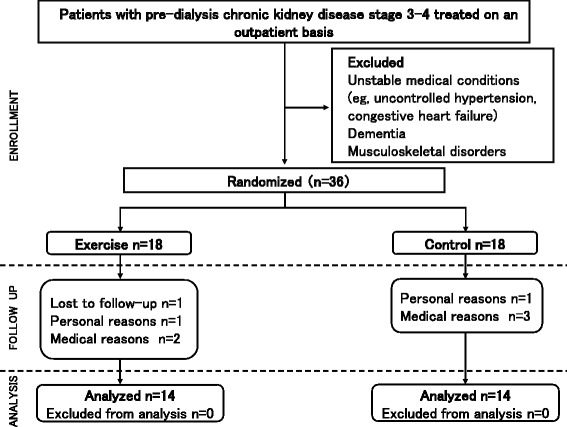
Flow of patients through the study

The subjects were recruited from August, 2011 to January, 2013.Thirty-six subjects (age, 68.7 ± 6.8 years; estimated glomerular filtration rate (eGFR), 39.0 ± 11.6 ml/min/1.73 m^2^) were included. The subjects were stage 3–4 pre-dialysis CKD patients who were being treated as outpatients (Fig. 1). The inclusion criterion was stage 3–4 CKD with stable kidney function. In order to exclude sex-based differences in muscle strength [12], only male patients were selected. Patients with the following conditions were excluded: uncontrolled hypertension and cardiac failure, motor disorders, and dementia. The study details were explained to the subjects, and consent was obtained for participation in the study.

### Design of randomization

The subjects were randomly allocated to an exercise intervention (Ex group) or control (C group) group by using a computer-based table of random numbers with treatment allocation ratio of 1:1. The allocation conditions were stratified according to the presence or absence of diabetes and eGFR values but blocking strategy was not adopted. Assignment was not blinded.

### Interventions

The Ex group performed home-based aerobic and resistance training exercises without supervision for a period of 1 year after they were instructed how to do it at first visit in the study period. Exercise which patients actually performed were collected from patients during outpatient visits every 2–3 months, and feedback was provided at each visit.

The aerobic exercises were in accordance with the exercise guidelines in Japan [13]. The Ex group was instructed to perform exercise activities, such as brisk walking for 30 min a day or completing 8000–10,000 steps per day. The amount of physical exercise performed was measured by using a Kenz Lifecorder EX 1-axial accelerometer pedometer with an acceleration sensor (Suzuken Co Ltd., Nagoya, Japan). The accelerometer pedometers were worn continuously for 1 year and were removed only when bathing or sleeping [14]. The physical exercise indicators were number of steps (steps/day) [14], amount of exercise performed (total amount of calories burnt through exercise: kcal/day) [14], and time spent on performing mid-level load exercises (min/day) [15].

Resistance training involved the use of a handgrip strengthening device for exercising the upper limbs and mid-level load exercises such as squats and calf raises for exercising the lower limbs [16]; subjects performed 20–30 repetitions per exercise, a minimum of 3 times per week. We gave everyone in the exercise group a handgrip strengthening device. The Ex group was trained to use an exercise record sheet to report adherence to resistance training, and the resistance training implementation rate and exercise details were derived from data in this sheet. Both the aerobic exercise and the resistance training used the Borg scale [17], which is a rating of perceived exertion (RPE), as the objective to establish a mid-level load strength.

The C group subjects also wore accelerometer pedometers for a period of 1 year, but these subjects were not given any exercise instructions and were asked to carry out daily activities as usual. The total number of steps recorded for the C group subjects was evaluated during the follow-up visits, and additional information related to exercise was not collected.

### Outcomes

Primary outcome was changes in muscle strengths (handgrip ad knee extensor muscle), and secondary outcome was changes in kidney functions by eGFR and urinary protein, both between baseline and after 12 months. Feasibility was assessed by changes in physical activity between baseline and after 12 months.

### Measuremenets

Baseline data collected from electric medical records included age, body mass index, presence of diabetes, eGFR, urinary protein per gram creatinine (UP: g/gCr), and hemoglobin and serum albumin levels.

The eGFR (ml/min/1.73 m^2^) [18], which was calculated from the serum creatinine and urinary protein levels, was used as the kidney function index. The pre- and post-intervention eGFR and UP levels were compared in both groups.

Muscle strength was measured as an indicator of physical function. Upper and lower limb muscle strength was determined by measuring handgrip and knee extensor muscle strength, respectively, by using a method described in a previous study [4]. Handgrip strength was measured by using a grip meter (JAMAR® hand dynamometer, Bissell Healthcare Co., Grand Rapids, MI); two measurements each were taken for the left and right arms, with the patient in a seated position with elbows bent to 90 degrees and forearms in mid-position, and the highest value was recorded. The handgrip strength (kgf) was defined as the average value for the left and right hand. Isometric knee extensor muscle strength was measured by using a hand-held dynamometer (μTAS MF-01®, Anima Inc., Tokyo, Japan); maximum muscle strength was measured twice each for the right and left legs, and the knee extensor muscle strength was determined by dividing the average value for the left and right leg by body weight (kgf/kg). As a secondary outcome, the change in handgrip strength and knee extensor muscle strength between T1 and T2 was compared between both groups.

### Statistical analysis

Each indicator was expressed as the mean ± standard deviation (SD). The chi-square test and non-paired t-test were used to compare baseline patient data between the two groups. In order to compare pre- and post-intervention changes in physical function, kidney function, and arm and leg muscle strength, we performed two-way factorial analysis of variance on two factors: group (presence or absence of intervention) and time (T1 and T2). Furthermore, the rate of improvement in muscle strength was calculated as follows: (T2 – T1)/T1. The non-paired t-test was used to compare the rate of improvement in muscle strength between the groups. Analysis was conducted using IBM SPSS ver.17.0 J for Windows (IBM SPSS Japan, Inc., Tokyo, Japan). Statistical significance was set at below 5%.

## Results

### Baseline characteristics

After the random allocation, both the Ex and C groups included 18 subjects each (Fig. 1). However, during the follow-up, 4 subjects dropped out from each group. The reasons for the drop-outs in the Ex group were as follows: did not agree to follow-up visits [1], job constraints [1], and hospitalization for other conditions (cancer, 1; stroke, 1). The reasons in the C group were: job constraints [1] and hospitalization for other conditions (cancer, 1; stroke, 1; and dizziness, 1). As a result, 28 subjects were included in the final analysis (Ex group: 14; C group: 14).

The baseline patient characteristics are shown in Table 1. No difference was observed between most variables; however, the C group tended to be more physically active than the Ex group. There was no significant differences in the baseline characteristics of the 8 patients who dropped out (data not shown).

**Table 1:** Baseline Patients Characteristics

	ALL *n* = 28	Exercise *n* = 14	Control *n* = 14	t or χ 2	*P*
Age(y)	68.5 ± 6.5	69.0 ± 6.8	67.8 ± 6.9	0.4	0.67
BMI (kg/m^2^)	23.7 ± 3.1	24.4 ± 3.5	23.0 ± 2.5	1.2	0.25
Cause of CKD, *N*				3.3^a^	0.50
Nephrosclerosis	14	7	7		
Chronic glomerulonephritis	8	4	4		
Diabetic nephropathy	2	2	0		
Polycystic kidney disease	1	0	1		
Unknown	3	1	2		
eGFR (ml/min/1.73 m^2^)	39.5 ± 11.4	37.6 ± 11.1	41.5 ± 11.8	−0.9	0.38
Urinary protein (g/gCr)	0.9 ± 1.1	0.9 ± 1.0	0.9 ± 1.4	0.2	0.80
Hemoglobin (g/dL)	13.1 ± 1.5	12.6 ± 1.7	13.6 ± 1.1	−1.9	0.07
Serum albumin (g/dL)	4.0 ± 0.3	3.9 ± 0.3	4.0 ± 0.3	−0.9	0.38
Handgrip strength(kgf)	33.6 ± 8.2	31.7 ± 7.4	35.5 ± 8.8	−1.2	0.23
knee extensor muscle strength(kgf/kg)	0.66 ± 0.16	0.65 ± 0.17	0.66 ± 0.15	−0.2	0.86
Average daily number of steps(steps/day)	7919.1 ± 3401.1	6725.3 ± 3152.4	9113.0 ± 3319.2	−1.9	0.07
Average daily energy expenditure(kcal)	215.3 ± 113.0	178.6 ± 103.9	252.0 ± 113.3	−1.8	0.09
Average daily moderate exercise time(min/day)	26.4 ± 17.8	22.6 ± 15.9	30.5 ± 19.5	−1.1	0.26

Data are expressed as the means ± SD or number of patients

*Abbreviations*: *BMI* body mass index, *CKD* chronic kidney disease, *eGFR* estimated glomerular filtration rate, *SD* standard deviation

^a^χ^2^value

### Feasibility of the trial

The changes in physical activity levels before and after the intervention are shown in Table 2. The change in the number of steps from T1 to T2 in the Ex and C groups was 6725.3 ± 3152.4 to 8281.4 ± 3108.8 steps/day and 9113.0 ± 3319.2 to 8728.8 ± 2850.9 steps/day (F = 4.3, *p* = 0.04), respectively; the number of steps increased significantly in the Ex group only. The amount of exercise performed increased from 178.6 ± 103.9 to 237.6 ± 111.3 kcal/day in the Ex group and from 252.0 ± 113.3 to 240.9 ± 91.9 kcal/day (F = 5.3, *p* = 0.03) in the C group, demonstrating a significant increase only in the Ex group. The time spent on mid-level load exercise changed from 22.6 ± 15.9 to 34.6 ± 23.7 min/day in the Ex group to 30.5 ± 19.5 to 33.3 ± 21.0 min/day (F = 2.7, *p* = 0.09) in the C group, with a considerable increase being observed in the Ex group only. The number of daily steps taken in the control group was 26% higher than at baseline. However, the number of steps increased significantly only in the exercise group, and time spent on exercise after 12 months was nearly equal in both groups. The implementation rate for resistance training over the year averaged 70.4% in the Ex group, i.e., the subjects performed resistance training for 5 days per week. There were no serious adverse events related to the exercise training.

**Table 2:** Changes in Physical Activity after the 12-Month Period

	Exercise		Control		ANOVA	
	Baseline	12-Month	Baseline	12-Month	*F*	*P*
Average daily number of steps(steps/day)	6725.3 ± 3152.4	8281.4 ± 3108.8	9113.0 ± 3319.2	8828.8 ± 2850.9	4.3	0.04
Average daily energy expenditure(kcal/day)	178.6 ± 103.9	237.6 ± 111.3	252.0 ± 113.3	240.9 ± 91.9	5.3	0.03
Average daily moderate exercise time(min/day)	22.6 ± 15.9	34.6 ± 23.7	30.5 ± 19.5	33.3 ± 21.0	2.7	0.09

Exercise group, *n* = 14; Control group, *n* = 14. Data are expressed as the means ± SD

*Abbreviations*: *ANOVA* analysis of variance, *SD* standard deviation

### Primary outcome

The pre- and post-intervention changes in muscle strength are shown in Table 3. Handgrip strength changed from 31.7 ± 7.4 to 36.4 ± 6.4 kgf in the Ex group and from 35.5 ± 8.8 to 36.5 ± 9.2 kgf (F = 7.0, *p* = 0.01) in the C group; a significant improvement was observed only in the Ex group. Knee extensor muscle strength changed from 0.65 ± 0.17 to 0.70 ± 0.17 kgf/kg in the Ex group and from 0.66 ± 0.15 to 0.62 ± 0.13 kgf/kg (F = 14.3, *p* < 0.01) in the C group; a significant improvement was observed only in the Ex group (*p* = 0.02, *p* < 0.01).

**Table 3:** Changes in eGFR, urinary protein, Handgrip strength, Knee extensor muscle strength after the 12-Month Period

	Exercise			Control		
	Baseline	12-Month	Baseline	12-Month	*F or t*	*P*
eGFR (ml/min/1.73 m^2^)	37.0 ± 10.9	35.1 ± 11.4	41.1 ± 12.2	39.5 ± 12.9	0.01	0.93
Urinary Protein (g/gCr)	0.9 ± 1.0	1.2 ± 1.7	0.9 ± 1.4	0.9 ± 1.1	0.4	0.52
Handgrip strength(kgf)	31.7 ± 7.4	36.4 ± 6.4	35.5 ± 8.8	36.5 ± 9.2	7.0	0.01
Change(%)		17.0 ± 16.1		3.4 ± 11.2	2.6^a^	0.02
Knee extensor muscle strength(kgf/kg)	0.65 ± 0.17	0.70 ± 0.17	0.66 ± 0.15	0.62 ± 0.13	14.3	<0.01
Change(%)		8.2 ± 10.9		−6.0 ± 7.6	4.0^a^	<0.01

Values are the mean ± SD unless otherwise noted

*Abbreviations*: *eGFR* estimated glomerular filtration rate, *ANOVA* analysis of variance, *SD* standard deviation

^a^t value

### Secondary outcome

The pre- and post-intervention changes in the eGFR are shown in Table 3. The eGFR reduced slightly in both groups: from 37.0 ± 10.9 to 35.1 ± 11.4 ml/min/1.73 m^2^ in the Ex group and 41.1 ± 12.2 to 39.5 ± 12.9 ml/min/1.73 m^2^ (F = 0.01, *p* = 0.93) in the C group; however, there were no significant differences between the two groups.

## Discussion

This study showed that home-based exercise in patients with pre-dialysis CKD performed over a period of 1 year was feasible and that it improved the muscle strength in arms and legs, and had no positive or negative effect on the kidney function.

Although home-based exercises are regarded to be effective and have no associated costs, the lack of supervision results in disadvantages such as safety, adherence to the program, and incorrect quantification of exercise [19]. Therefore, we used accelerometer pedometers in order to quantify the amount of aerobic exercise performed. Previous research has shown that the use of pedometers alone leads to an increase in physical activity [20]. In our study, an increase in the number of steps and energy expenditure due to exercise was observed only in the Ex group and, additionally, the time spent on moderate intensity exercise increased from 22 to 34 min in the Ex group, all of which strongly supports the feasibility of home-based exercise therapy in this population. These findings also indicate that the use of the accelerometer pedometer could have been a motivator, resulting in the Ex group subjects performing moderate aerobic exercise for 30 min per day over a period of 1 year. Subjects were also required to use the exercise record sheets to perform self-checks on the frequency of resistance training. Subjects also performed resistance training regularly because they were asked about training adherence during the follow-up visits. Further, the exercise program consisted of exercises that could be incorporated into the daily lives of the subjects, making adherence easier. Interestingly, the level of physical activity was high in the C group right from the baseline. We believe that the C group consisted mainly of individuals who had a desire to exercise and were further motivated by the use of the accelerometer pedometer.

A previous study by Izawa et al. [16] reported increased muscle strength only in the group of myocardial infarction patients who engaged in home-based walking exercises with resistance training (squats and calf raises). In the present study, an increase in grip strength by 17.0% and knee extension muscle strength by 8.2% was observed only in the Ex group subjects who performed a combination of moderate walking and resistance training exercises; this indicates that home-based exercise therapy can improve muscle strength. However, it is difficult to set an objective load intensity for home-based resistance training. The subjects in this study used their own body weight as the load during resistance training, and we estimate that the load intensity ranged from light to moderate. In addition, we consider that the high mean monthly implementation rate of 70% contributed to the increased muscle strength. Nevertheless, as compared to previous interventional studies on resistance training for pre-dialysis CKD patients [1, 21], the rate of improvement in knee extension muscle strength in the present study was slightly lower. A systematic review of the effect of home-based resistance training on elderly subjects indicated that muscle strength improved, but the improvement was small [19]. Another consideration is the fact that the grip strength and knee extension muscle strength of the subjects in the present study were high at the baseline when compared to subjects in our previous study [4].

A previous research indicated that the rate of kidney function decline in elderly stage 3–4 CKD patients (mean age, 67 years) is −3.5 ml/min/year [22]. However, we found no difference in the mean eGFR values (1–2 ml/min/1.73 m^2^) between the two groups. This finding suggests that performing moderate exercise for a period of 1 year failed to show positive effect but did not either exerted negative effect on the kidney function of the pre-dialysis CKD patients. Studies have shown that eGFR either remains unchanged [23] or improves [24] in pre-dialysis CKD patients who engage in exercise therapy for a period of 1 year; therefore, there is no consensus on the effect of exercise on kidney function. A recent observational study that compared individuals who engaged in walking and other forms of exercise with those who did not engage in exercise activity indicated that patients who engaged in more physical activity had a lower rate of eGFR decline [25]. However, since the subjects in the present study had stable kidney function at the baseline, increased physical activity did not lead to improvements in eGFR. Greenwood et al. [24] reported improvements in eGFR; however, the subjects had a mean age that was 15 years lesser than that in our study and they excluded subjects who had a habit of exercising regularly. Further, in the present study, diet-related interventions were not strictly implemented. We believe that these factors contributed to the lack of exercise-induced improvement in kidney function. However, we calculated the eGFR values from serum creatinine levels [18]. The increase in arm and leg muscle strength in the Ex group could be related to an increase in muscle mass. Therefore, the eGFR levels calculated from serum creatinine levels, which are influenced by muscle mass, may not have specifically reflected the exercise-induced improvement in kidney function. Future studies using cystatin C and other indicators that are not affected by muscle mass would be beneficial.

This study had several limitations. First, the number of subjects was small. Since previous interventional studies on exercise therapy for pre-dialysis CKD patients were also small-scale studies, it is necessary to conduct large-scale, long-term interventional studies in the future. Second, this study was a single-institution study and neither the researchers nor the subjects were blinded. Third, the level of physical activity of the subjects in the C group was already high at the baseline. However, because the level of physical activity in the C group did not increase during the course of the study and there were no changes in kidney function or muscle strength, we believe that this did not have a major effect on the results of this study.

## Conclusions

Home-based exercise therapy for pre-dialysis CKD patients was feasible and improved arm and leg muscle strength.

## Abbreviations

CKD: Chronic kidney disease
eGFR: Estimated glomerular filtration rate
UP: Urinary protein
